# Sexual risky behavior, cocaine and alcohol use among substance users in an outpatient facility: a cross section study

**DOI:** 10.1186/s13011-019-0238-x

**Published:** 2019-11-06

**Authors:** Claudia Chaves Dallelucci, Emi Carneiro Bragiato, Kelsy Catherina Nema Areco, Thiago Marques Fidalgo, Dartiu Xavier da Silveira

**Affiliations:** 1 Psychiatry Department, Universidade Federal de São Paulo, Major Maragliano Street, 241, São Paulo, SP 04017-030 Brazil; 2 Health Informatics Department, Universidade Federal de São Paulo, São Paulo, SP Brazil

**Keywords:** Alcohol abuse, Cocaine abuse, Sexual behavior, Condom, Risk behavior

## Abstract

**Background:**

Substance dependence is considered an international health issue and studies that access the characteristic of this population are required to develop public health programs for harm reduction. To this aim, we purpose to to identify, in a population undergo addiction treatment, if the use of substances leads to sexual risky behavior and also check if another variables influences in this behavior.

**Method:**

Observational study of clinical sample collected of adult patients seeking treatment to dependence of alcohol and cocaine. The data collected were: socio-demographic, substance use, sexual behavior and childhood abuse. Risky sexual behaviors were considered: inconsistent use of condoms and presence of multiple sexual partners in the past six months. An exploratory analysis of the association between the variable “risky sexual behavior” and the other variables was performed using Pearson’s chi-square, followed by a multivariate logistic regression analysis.

**Results:**

After analyzing the variables with the presence or absence of risky sexual behavior, were included in the logistic regression the data that presented association with sexual risk behavior, and age appears as an independent factor for risky sexual behaviors. Other factors, such as schooling and unemployment, influence as much as the use of substances in risky sexual behaviors.

**Conclusion:**

Attributing just to the substance use this risky sexual behavior seems too simplistic. Other structural factors such as schooling, work, age and sexual abuse in childhood can influence decision making for safe sex.

## Background

The use of psychoactive substances in not something new, it has been present throughout the history of mankind [1]. When the user develops dependence, the use of drugs becomes extremely harmful because there is a strong compulsion for the substance, and there may be symptoms of tolerance and abstinence, abandon of other pleasures or interests and the continuance of the use even when the user is aware of its harmful effects [2]. Nowadays, substances dependence is considered an international public health issue which causes severe consequences to both user and society [3] and studies that access the characteristic of this population are required to develop public health programs for harm reduction. This is important to all countries that face drug related problem, but it is urgent among low and middle-income countries. This group of countries have scare funding and the investments must be evidence-based.

According to the III Household Survey on the Use of Psychotropic Drugs in Brazil, in 2017, in Brazil the use of alcohol at least once in a person’s lifetime is 66.4%. Among them, 3.5% meet criteria for dependence of alcohol. Regarding cocaine, inhaled or smoked, 3.1% of the interviewed subjects have used it at least once in their lives and 7.7% have used marijuana. In addition, 4.8% of the individuals use alcohol and another substance. The use of alcohol and illicit substances is higher among the male population and there are significant variations as to school background and age, depending on the drug that has been analyzed [4]. The association of more than one substance is a usual practice among drug users and the most common association use of cocaine with alcohol [5] which forms a chemical compound named cocaethylene, with more potent effects of intoxication and also more health damage [6].

The use of both legal and illegal drugs has several consequences with negative results for the individual’s social, personal and professional life, besides increasing health related expenses. One of the harmful results is the increase of unsafe sexual practices, mainly the practice of sex without condom resulting in a higher risk of contracting sexually transmitted infections [7, 8]. Moreover, some authors also mention prostitution as a way to support the use, a practice which may ultimately lead to unprotected sexual intercourse [9].

The exact modulating effect of the psychoactive substances on sexual practices has not been totally clarified yet. However, it has been suggested that the use of drugs may be considered an independent predictor of the unprotected sexual practice [10]. Furthermore, there is a relation between the drug effects and the sexual activity, such as libido increase, changes in body sensations, and decrease in social inhibitions. The decrease in reflexes and planned choices associated with the use of substances may lead to risky sexual intercourse [8, 9, 11].

Some authors suggest that several harmful behaviors may occur together, which increases risks to health [12, 13] and these risks are mediated by structural causes other than the use of psychoactive substances [14–16]. According to Jessor & Jessor’s behavioral issue theory, the involvement of a risky behavior increases the likelihood of other risky behaviors, leading to a cycle of activities potentially harmful to the individual’s physical and psychological integrity [17].

This research addresses the relation between the use of psychoactive substances and risky sexual behavior. In their first interview some patients who undergo dependence treatment describe risky sexual behavior. The current study intends to address the association between risky sexual behaviors – mainly the inconsistency in the use of condom in combination with the presence of multiple partners – and the use of alcohol and alcohol associated with cocaine, in an attempt to determine if the combined use of drugs produces an addictive effect over risky sexual behaviors. Also, we considered studying which social factors, together with substances use, interfere in the safe sexual practices in this sample.

## Method

### Study outline

The current research is an observational study of clinical sample, based on data collected from the standard electronic records used during the first interview conducted with patients who search for an Addiction Unit (PROAD, in the Portuguese acronym) for drugs dependence treatment.

### Sample

The studied sample is one of convenience, comprehending all adult patients from both sexes who searched for PROAD (Addiction Unit of the Department of Psychiatric – Universidade Federal de São Paulo - UNIFESP) for treatment of their substance dependence, from January 2008 to May 2016. Only those with moderate or severe dependence of alcohol or cocaine or both according to DSM-IV (diagnostic and statistical manual of mental disorders) were included. Although part of data collection was performed before the release of DSM-IV criteria of dependence, the records of the patients had enough information for us to stablish their diagnosis according to DSM-IV. The use of tobacco was not considered an exclusion criterion.

The following excluding criteria were consiered: (1) Records with unanswered sexual behavior questionnaire; (2) Patients who declared not having practiced sex in the last six months.

From the total amount of 711 patients records in the period, 318 files were of patients with exclusive use of alcohol and alcohol with inhaled or smoked cocaine were selected for this study, and the final sample comprised 245 individuals after checking the excluding criteria, which corresponds to 77% from the selected records.

Considering the outcome “sexual risky behavior” in the two groups (alcohol users and alcohol + cocaine users), the total simple size (*N* = 245), the proportion of the outcome in each group (10 and 20%), significance level of 5% and sampling ratio 2.5 (175/70), the sample power was 94%. Considering the association analysis between the outcome and other variables (alcohol and alcohol + cocaine groups and covariates), the sample size was close to *N* = 100 + 20i (i = 7, counting the numerical and dichotomous as variable 1 and the number of dummy variables (number of categories − 1) for categorical variables with more than two categories). The most recommended formula is N = 100 + 50i [18].

### Data collecting procedure

The first procedure at the institution is patient screening, with only the patient and the screener where the individualized therapeutic planning is carried out. It is a standard structured interview, with the use of instruments following an order, conducted by doctors or nurses, which lasts nearly one hour.

The main instruments used at the first appointment and in the current study address socio-demographic characteristics, identification of sexual abuse in childhood, practices related to sexual behavior and identification of psychoactive substances used in the last year.

The patients undergo the treatment voluntarily and the collected data is confidential. The screener explains that neither answering the questions presented in the instruments nor undergoing treatment is mandatory and the patients are free to stop the interview at any moment they wish to do so. Since the institution is devoted to conducting study and research, at the first interview the patients are informed that their authorization concerning the use of the unidentifiable data may be requested, provided such data is useful for a specific clinical study. It is important to highlight that the treatment is in no way harmed by the patients’ refusal in any phase.

### Variables

The variables of the present study were organized in three blocks: socio-demographics, substance use, and sexual behavior.

#### Socio-demographic data

The following socio-demographic data available at the screening were selected: age, sex, marital status, schooling and working.

Schooling was divided in a scalar non-dichotomized variable, the categories being “illiterate or up to 8 years of study”, “9 to 12 years of study” and “over 12 years of study”. Although unbalanced in number of years studied, this categorization was used due to data dispersion, so that no category had much more subjects than the others. As to working the results were categorized dichotomously with the possible answers being “yes” and “no” to the question whether the patient was practicing remunerated activity when the screening was conducted. Marital status was categorized as “single” and “married”; patients who identified themselves as widows/widowers and separated were included in the single category; the ones who lived with their partners out of wedlock were considered married.

#### Use of substances

The instruments include questions on use of substances and were developed at PROAD based on the questionnaire issued by the World Health Organization (WHO) in 2008 to assess the use of drugs at primary care [19]. The questionnaire presents the possible answers “yes” or “no” to the use in the past year and the affirmative answer for more than one substance is possible. According to the use of substances in the past year, the users were classified in two groups: exclusive use of alcohol and use of alcohol with inhaled or smoked cocaine.

#### Sexual behavior and sexual abuse in childhood

The current study selected two questions to analyze sexual risk behavior: “how many people have you had sex in the last six months?” and “how frequent have you worn condom in the last six months during sexual intercourse?”, which led to the possible answers: “never”, “few times”, “most of the times” and “always”. Such answers were afterwards dichotomized as “inconsistent use” versus “consistent use”. Based on this two information a new variable was created to analyze the composite outcome, considering as risky sexual behavior in the past six months just the inconsistency in the use of condom and the sexual practice with more than one partner in the past six months.

Besides the above mentioned questions the following were included in the analyses: “have you ever had sex for money or drugs?”, “in the last six months have you had sex under the effect of alcohol or other drugs?” and “did you suffer any kind of sexual abuse in childhood?”

It is important to state that these questions were developed by the Addiction Unit facility and they do not comprise a validated instrument developed to make an in-depth analysis of risk sexual behaviors. All questions were self-reported. We believe that self-report was the best way to obtain these information, as sexual behavior and history of sexual abuse might be sensitive topics for most people and the presence of an interviewer could have led the subjects to not give the correct answers, in order to avoid judgments.

### Data analysis

According to the aim, the study conducted the exploratory analysis of the association between the outcome variable “risky sexual behavior” and the group of substances users (alcohol exclusively and alcohol with inhaled or smoked cocaine) and the other variables, followed by a multivariate analysis.

A descriptive statistical analysis of the variables according to their nature, numeric or categoric, was carried on throughout the sample. The categoric variables were described by relative and absolute frequency, and the numeric variable “age” (in years) was described by central tendency measures (mean, medium) and variability (minimum, maximum, standard deviation, interquartile range) and tested in relation to adhesion to normal distribution by the Kolmogorov-Smirnov test.

The study on the association between risky sexual behavior and the group of users and the other categoric variables was conducted through the Pearson’s chi-square test (*X*^*2*^). In order to study the association between the numeric variable age and the outcome variable “risky sexual behavior” the Mann-Whitney *U* test was conducted, since “age” was not normally distributed between the two outcome categories.

The multivariate analysis was chosen as logistic regression analysis [20]. The selection of the variables to compose the logistic regression model was based on the results obtained by the exploratory analysis: the variables selected were those which presented descriptive level (*p* value) inferior to 0.20 during the association test. To ensure the absence of association between the independent variables, thus avoiding multicollinearity in the model, Cramer’s *V* test was conducted. The test was interpreted according to Pett [21]. No variable presented values superior to 0.50 to that coefficient (data not shown).

Once selected the explanatory variables, the univariate logistic analysis was conducted, presenting the Odds Ratio (*OR*) values, *p* value and confidence interval (*CI* 95%). Subsequently was carried on multivariate logistic regression, obtaining adjusted Odds Ratio (*OR*^*a*^), adjusted *p* value (*p* value^a^) and adjusted confidence interval (*CI* 95%^a^).

The program SPSS 22.0 was used throughout the analysis in all the phases described above; the relevancy level of 5% was established for all the tests.

### Ethical aspects

The study project was assessed and approved by Research Ethics Committee of ‘Universidade Federal de São Paulo’ (Protocol n. ° 0798/2016). The active patients included in the study received the information about it and signed the Informed Consent Form. Three attempts were made to contact by phone patients who were no longer undergoing treatment and they were informed of the present study and invited to sign the form if they wished to do so. Nevertheless, since it was necessary to work retroactively in revising patients’ registers the contact was not successful. In such cases the Ethical Committee authorized the use of data without the Informed Consent Form, but the researchers were compromised to keep privacy of the collected data remained. Considering the likely vulnerability of the patients and also their possible involvement in potential illegal activities, the data bank as well as the medical records consultation were made anonymous and secret.

## Results

Among the 245 medical records included in this analysis, 235 (95.9%) were from men and 10 (4.1%) from women. The average age was 37.83 years (SD (Standard deviation): 11.17; Min: 18; Max: 74). Most of the sample subjects (40.0%) ranged between 9 and 12 years of study, 35.1% reported over 12 years of studying and 24.9% reported none or less than 8 years. As to marital status, 76.7% were married and 23.3% were single. 56.1% were working in the beginning of the treatment, while 43.9% did not practice any remunerated activity.

Besides the risky sexual behavior, present in 16.7% of the sample and absent in 83.3%, other questions addressing sexual behavior were included, 10.2% had sexual intercourse in exchange for money or drugs while 89.9% did not engage in such behavior. 71.4% have had sexual intercourse under the influence of alcohol or drugs in the last six months and 28.6% did not report such behavior. 13.9% reported having suffered sexual abuse in childhood and 81.6% denied it.

The presence of use of substances the year before identified that 52.2% use alcohol exclusively while 47.8% used alcohol with inhaled or smoked cocaine (Table 1).

**Table 1:** Socio-demographic characteristics, use of alcohol exclusively or associated with cocaine use, sexual practices and history of sexual abuse and their association with risky sexual behavior. PROAD, São Paulo, Brazil, 2008 to 2016 (*N* = 245)

Variables				Risky Sexual Behavior				
		TOTAL		YES		NO		*P* value
*Age* *N: 245*	*Mean*	*37,83*		*33,76*		*38,65*		*0,008*
	*Median*	*37,00*		*33,00*		*38,00*		
	*SD*	*11,17*		*10,42*		*11,16*		
	*Min*	*18*		*20*		*18*		
	*Max*	*74*		*64*		*74*		
	*Interquartile range*	*16*		*14*		*16*		
Total		N	%	N	%	N	%	
		245	100	41	16,7	204	83,3	
Sex N: 245	Male	235	95,9	38	16,2	197	83,8	0,251
	Female	10	4,1	3	30,0	7	70,0	
Schooling N: 245	No study or until 8 years of study 9 a 12 years of study More than 12 years of study	61	24,9	9	14,8	52	85,2	0,130
		98	40,0	22	22,4	76	77,6	
		86	35,1	10	11,6	76	88,4	
Working N: 244	No	107	43,9	23	21,5	84	78,5	0,083
	Yes	137	56,1	18	13,1	119	86,9	
Marital Status N: 245	Married	188	76,7	31	16,5	157	83,5	0,852
	Single	57	23,3	10	17,5	47	82,5	
Drug use in the past year N: 245	Exclusive alcohol use	128	52,2	17	13,3	111	86,7	0,130
	Alcohol with cocaine/crack use	117	47,8	24	20,5	93	79,5	
Have you ever had sex for money or drugs? N: 245	No	220	89,8	35	15,9	185	84,1	0,304
	Yes	25	10,2	6	24,0	19	76,0	
In the last six months, have you had sex while under effect of drugs or alcohol? N: 245	No	70	28,6	7	10,0	63	90,0	0,074
	Yes	175	71,4	34	19,4	141	80,6	
Have you suffered sexual abuse in childhood? N: 245	No	211	86,1	32	15,2	179	84,8	0,101
	Yes	34	13,9	9	26,5	25	73,5	

Afterwards, an analysis of the categoric variables was conducted according to the presence or absence of risky sexual behavior. The average age among those who presented risky sexual behavior was 33.76 (SD: 10.42; Min: 20; Max: 64), and among those who did not present risky sexual behavior the average age was 38.65 (SD: 11.16; Min: 18; Max: 74) (*p* = 0,008). 16.2% of men and 30% of women reported having practiced risky sexual behavior (*p* = 0,251). Among the patients who presented risky sexual behavior 14.8% completed less than 8 years of study, 22.4% ranged between 9 and 12 years of study, and 11.6% completed more than 12 years of study (*p* = 0,130). As to working, among those who engaged in risky sexual behavior 13.1% were employed and 21.5% were unemployed (*p* = 0,083). Among married subjects 16.5% reported having risky sexual behavior and among the single subjects 17.5% admitted having risky sexual behavior (*p* = 0,852).

The risky sexual behavior is present in 13.3% of subjects who used alcohol exclusively and in 20.5% of those who used alcohol with inhaled or smoked cocaine. On the other hand, the same behavior is absent in 87.7% of subjects who used alcohol exclusively and in 79.5% of those who used alcohol with inhaled or smoked cocaine (*p* = 0,130).

About the question “have you ever had sex for money or drugs?”, 24% of the subjects who showed risky sexual behavior answered “yes”, while 15.9% answered “no” (*p* = 0,304). When asked “have you had sex under the effect of alcohol or drugs in the last six months?” 19.4% of those subjects who had sex under the influence of substances had risky sexual behavior, while the same practice is present in 10% of those who did not have sex under the influence of substances (*p* = 0,074). Among those subjects who suffered sexual abuse in childhood 26.5% presented risky sexual behavior and among those who denied having suffered sexual abuse in childhood 15.2% presented risky sexual behavior (*p* = 0,101) (Table 1).

The following independent variables were considered in the multiple logistic regression: age, schooling, unemployment, use of cocaine with alcohol, practice of sex under the effect of substances in the last six months, and history of sexual abuse in childhood. The results showed that age is an independent factor associated with risky sexual behavior, each year added to the subjects’ age progressively decreases the risk for such behavior. The group of subjects who completed more than 12 years of study also presented less probability of engaging in risky sexual behavior. The other variables did not show statistically significant association with the risky sexual behavior (Table 2).

**Table 2:** Uni and Multivariate Logistic Regression: age (in years), schooling degree, unemployment, use of alcohol with inhaled or smoked cocaine in the past year, having sex under the effect of drugs, history of sexual abuse in childhood related to risky sexual behavior. PROAD, São Paulo, Brazil, 2008 to 2016 (*N* = 245)

Variables	Univariate Logistic Regression			Multivariate Logistic Regression		
	OR^*a*^	95% CI^*b*^	P value	^a^OR^*c*^	^a^95% CI^*d*^	^a^*P* value^*e*^
Age, Years	0,957	0,926-0,990	0,012	0,965	0,993-0,999	0,045
Schooling						
*None or until 8 years of study*	1	–	0,137	1	–	0,121
*9 a 12 years of study*	1,315	0,500-3,460	0,579	1,659	0,594-4,635	0,334
*More than 12 years of study*	2,200	0,976-4,957	0,057	2,446	1039-5,763	0,041
Working at the time, No	1,810	0,920-3,563	0,086	1,809	0,888-3,686	0,102
Alcohol with cocaine/crack use in the past year	1,685	0,854-3,325	0,132	1,635	0,806-3,318	0,173
Sex under effect of drugs, Yes	2,170	0,903-5,160	0,080	2,067	0,839-5,097	0,115
Sexual abuse in childhood, Yes	2,014	0,861-4,710	0,106	2,002	0,815-4,917	0,130

^*a:*^ Odds Ratio; ^*b:*^ Confidence interval; ^*c:*^ adjusted Odds Ratio; ^*d:*^ adjusted confidence interval; ^*e:*^ adjusted *p* value

## Discussion

As for sex, women comprised only 4.1% of our sample. A multicentric Brazilian study found that the percentages of women seeking for treatment varies from 11.3 to 19.3%, being higher at facilities with a specific program for women [22]. There are some possible explanations for this phenomenon. According to the III Household Survey on the use of psychotropic drugs in Brazil, there is a smaller number of women dependent on substances when compared to the male population, which might explain the smaller number of women seeking treatment for dependence [4]. Besides that, some studies suggest there may be other factors related to such difference. When we analyze the socio-cultural context we find out that the use of psychoactive substances by men is more tolerated than the use by women and the stigma caused by dependence may be a protective factor for women [23]. Furthermore, studies suggest women take longer to seek treatment, doing it when they find themselves in a more advanced level of dependence [24]. Some studies reveal that women are more likely to abide to moral and social laws, explaining a higher tendency to become dependent on prescribed substances [4, 23, 25].

The current study consistently shows the “age” associated to risky sexual behavior in substances users. However, the influence of age over the adult population is little discussed in the literature. Most of the studies on risky sexual behavior are conducted among adolescents, considering the exposure to risks intrinsic at the youngsters [26].

According to the result found by the multivariate analysis the variables “unemployed”, “having sex under the effect in the last six months”, “history of sexual abuse in childhood” and the use of alcohol with cocaine do not represent statistically significant factors. Nonetheless, this negative result may be due to the limited size of the available sample comprehending subjects engaged in risky sexual behavior (16.7%). Future studies may find associations in a larger sample.

The tendency to engage in risky sexual behavior is higher when the individual is unemployed. This datum, however, is little explored in the literature. We have not observed a statistically significant association between low educational levels and risky sexual behavior.

Both variables (unemployment and low educational level) are likely to be part of the same scope of causes leading to higher risks globally, including use of substances and inconsistent use of condom [17, 27, 28]. The use of illegal substances may be directed associated with low educational levels [29] and studies report that higher social-economic status, higher educational levels and employment lead to a higher use of condom [30, 31]. Structural factors, such as economic and social factors create and recreate risks; thus, the identification of possible psychosocial vulnerability profiles is mandatory for planning the care of the population with lower educational levels and less formal job opportunities [32, 33].

We found no association between history of sexual abuse in childhood and risky sexual behavior. There are some studies on association of sexual abuse during childhood with multiple disorders in adulthood, such as use of tobacco and alcohol, HIV (human immunodeficiency virus), STI (sexually transmitted infections), prostitution and eating disorders [34]. Considering that traumatic events in childhood seems to be related to a higher risk of developing substances dependence, psychiatric diseases and anti-social behavior in adulthood, risky sexual behavior seems to be another harmful consequence that sexual abuse may have [35]. In Brazil, a recent study with a sample of adults under treatment for substance use disorders found that 31.3% of them had a history of sexual abuse. The authors were able to conclude that being a victim of sexual abuse during childhood is a predictor of sexual risk behavior in adulthood [36].

Sexual intercourse under the influence of substances is related in the literature to risky sexual behavior and also choosing multiple partners and possible inconsistence in the use of condom [7, 10, 37]. Although this practice was not assessed as main outcome, it showed tendendy to association and a further study on this risky behavior would be necessary to consistently corroborate such association.

Some authors suggest the use of crack and cocaine would be an independent predictive factor of unprotected sex [7] and that, no matter the drug chosen, the use of any substance would be the main variable associated to the risk of contracting an STI [9], but such direct association was not clearly verified on the current study where we analyzed the profile of patients dependent on substances. The use of inhaled or smoked cocaine together with alcohol did not show statistically relevant worsening of the exposure to sexual risk; both groups presented similar probabilities; therefore, it cannot be considered an independent risk factor. One study revealed the association between the use of substances (except marijuana) with a larger number of partners, being the stronger association with use of cocaine. However, alcohol was the drug most related to the inconsistent use of condom [16].

Another researcher showed that the number of psychoactive substances used and the use of drugs during sexual intercourse did not alter the frequency in the use of condom, though there was an increase in the number of sexual partners [38]. Another study suggests alcohol as responsible for a higher risk of unplanned sex, but not for presence of multiple partners nor inconsistent use of condom [39]. Most of the current studies do not assess the inconsistent use of condom as related to the presence of multiple partners which may account for the significant difference in results obtained by several researches. It is important to highlight that most of the studies, including this one, did not take into consideration the standard use of the psychoactive substance nor the places where the substance is used, which may influence in the decision to have sex [40].

One of the strongest points of the research is the sample homogeneity obtained by data collection standards, since the patients came from the same addiction unit and went through the same screening protocol. Notwithstanding, there are limitations. The sample were selected by convenience, collected from records of patients spontaneously seeking for treatment in an outgoing addiction unit and the results may not be reliable to all dependent population in Brazil. Another limitation was the small proportion of women included. This made it impossible to find sex differences on the studied behaviors, which explains why this variable was not included in the regression model. This information is important, as some behaviors may be strongly influenced by cultural aspects of being a woman, such as negotiating condom use [41]. In addition to that, there were many statistical tests done with no adjustments for the number of tests. Thus, the chance for a Type I error might have been inflated. Finally, the object of this study is of personal and potential illegal nature and may have led to subnotification by biased information especially because it refers to the initial interview [42].

Like other studies addressing the association of the use of substances and risky sexual behavior, we verified that establishing a direct association of these factors seems to be a very simplistic way to discuss such complex human behaviors, mediated by several factors and present in different social and individual contexts. Some authors suggest there are personality traits which would affect both risks [43]. Besides, this is a transversal study and such affirmation may create reverse causality bias since this outline does not enables us to maintain that the use of substance precedes the risky sexual behavior. A study that tried to correct this bias by analyzing adolescents’ diaries shows that neither the use of substances nor the changing of partners alters the habit of condom’s use among this population. The study found a higher use of condom when there is sexual intercourse with a stable partner even under the influence of substances [42].

## Conclusion

Considering that both factors studied among the drug dependent population - the use of condom and multiple sexual partners – determine a possible HIV and STI transmission, it is safe to say the population above mentioned runs a high risk of contracting infections related to sexual practice. Yet, the results indicate that factors such as age, work status, schooling and history of sexual abuse, impact the risks those individuals are exposed to and it is controversial and difficult to prove that risky sexual behaviors are caused solely by the effect of use of substances. Addiction units should address sexual risk behaviors and, likewise, STI treatment units should provide counseling on the use of substances.

## Data Availability

The data will not be shared, considering the likely vulnerability of the patients and also their possible involvement in potential illegal activities, the data bank as well as the medical records consultation were made anonymous and secret, due solicitation of the Ethical Committee.

## Abbreviations

CI 95%: Confidence interval
DSM-IV: Diagnostic and statistical manual of mental disorders, Fourth Edition-TR
HIV: Human Immunodeficiency virus
OR: Odds Ratio
SD: Standard Deviation
STI: Sexually Transmitted Infections
WHO: World Health Organization
